# Use of a Primary Epithelial Cell Screening Tool to Investigate Phage Therapy in Cystic Fibrosis

**DOI:** 10.3389/fphar.2018.01330

**Published:** 2018-11-28

**Authors:** Stephanie Trend, Barbara J. Chang, Mark O’Dea, Stephen M. Stick, Anthony Kicic, WAERP, AusREC, AREST CF

**Affiliations:** ^1^Telethon Kids Institute, Perth, WA, Australia; ^2^Division of Paediatrics, School of Biomedical Sciences, The University of Western Australia, Perth, WA, Australia; ^3^The Marshall Centre for Infectious Diseases Research and Training, School of Biomedical Sciences, The University of Western Australia, Perth, WA, Australia; ^4^School of Veterinary and Life Sciences, Murdoch University, Perth, WA, Australia; ^5^Department of Respiratory Medicine, Perth Children’s Hospital, Perth, WA, Australia; ^6^Centre for Cell Therapy and Regenerative Medicine, School of Medicine and Pharmacology, The University of Western Australia and Harry Perkins Institute of Medical Research, Perth, WA, Australia; ^7^Occupation and the Environment, School of Public Health, Curtin University, Perth, WA, Australia

**Keywords:** cystic fibrosis, phage therapy, preclinical models, airway epithelial cells, *Pseudomonas aeruginosa*, infection

## Abstract

Antimicrobial-resistant microbes are an increasing threat to human health. In cystic fibrosis (CF), airway infections with *Pseudomonas aeruginosa* remain a key driver of lung damage. With few new antibiotics on the development horizon, alternative therapeutic approaches are needed against antimicrobial-resistant pathogens. Phage therapy, or the use of viruses that infect bacteria, is one proposed novel therapy to treat bacterial infections. However, the airways are complex microenvironments with unique characteristics that may affect the success of novel therapies. Here, three phages of *P. aeruginosa* (E79, F116, and one novel clinically derived isolate, designated P5) were screened for activity against 21 *P. aeruginosa* strains isolated from children with CF. Of these, phage E79 showed broad antibacterial activity (91% of tested strains sensitive) and was selected for further assessment. E79 genomic DNA was extracted, sequenced, and confirmed to contain no bacterial pathogenicity genes. High titre phage preparations were then purified using ion-exchange column chromatography and depleted of bacterial endotoxin. Primary airway epithelial cells derived from children with CF (*n* = 8, age range 0.2–5.5 years, 5 males) or healthy non-CF controls (*n* = 8, age range 2.5–4.0 years, 4 males) were then exposed to purified phage for 48 h. Levels of inflammatory IL-1β, IL-6, and IL-8 cytokine production were measured in culture supernatant by immunoassays and the extent of cellular apoptosis was measured using a ssDNA kit. Cytokine and apoptosis levels were compared between E79-stimulated and unstimulated controls, and, encouragingly, purified preparations of E79 did not stimulate any significant inflammatory cytokine responses or induce apoptosis in primary epithelial cells derived from children with or without CF. Collectively, this study demonstrates the feasibility of utilizing pre-clinical *in vitro* culture models to screen therapeutic candidates, and the potential of E79 as a therapeutic phage candidate in CF.

## Introduction

Cystic fibrosis (CF) is the most common autosomal recessive lethal genetic disease of Caucasian populations (Tian et al., 2016). In the lung, loss of CFTR function leads to production of viscous mucus, which is difficult to clear by standard mucociliary action (Koehler et al., 2004). This in turn contributes to the development of chronic infections with opportunistic bacterial pathogens such as *Pseudomonas aeruginosa* (Friman et al., 2013). The severity of lung bronchiectasis, excessive lung inflammation in children, and rate of decline in lung function in infants with CF is directly correlated with airway infections with *P. aeruginosa* (Koehler et al., 2004; Farrell et al., 2009; Pillarisetti et al., 2011).

Current therapies for CF lung infections include intravenous or inhaled antibiotics (National Guideline Alliance, 2017). However, antibiotic-resistant bacterial strains have emerged as major causes of mortality in hospitals worldwide, and in Western Australia, approximately 30% of *P. aeruginosa* isolates from CF patients are carbapenem-resistant (Tai et al., 2015). The World Health Organization has recognized antibiotic resistance as a significant threat to human health requiring urgent action (World Health Organization, 2014). Bacteriophages (phages) are viruses that infect bacteria, and present a novel treatment option in CF, however, little has been investigated regarding their potential in this setting. Concomitant with this need for new antibacterial treatments is the need for relevant *in vitro* models to screen potentially therapeutic phages and facilitate understanding of how these preparations act in the context of the human airway (Trend et al., 2017). Phage therapy holds enormous potential benefit for people with CF, and may complement existing antimicrobial strategies, since phages can replicate at the site of infection inside the target bacterial cells and subvert existing antimicrobial resistance in bacterial pathogens (Alemayehu et al., 2012; Sahota et al., 2015).

While the investigation and development of phage-derived products as therapeutic agents requires employment of *in vitro* antimicrobial assays, the effectiveness of any antimicrobial therapy *in vivo* may not always correspond to expected outcomes, due to a range of human factors not considered in the models (Henry et al., 2013). For example, host immune mediators directly interact with phage particles to inactivate them *in vivo* (Majewska et al., 2015). Furthermore, the patient’s innate immune system may induce an inflammatory response upon exposure to the phage, particularly if the phage preparations derived from culture in bacterial hosts are inadequately purified. Relevant *in vitro* models are thus essential in order to elucidate and understand the responses of the airway to this new potential therapy.

To develop phages for clinical trials, researchers should characterize and screen candidates for antimicrobial activity and carriage of bacterial pathogenicity genes. Given the inflammatory nature of many bacterial-derived products, stringent purification steps must be applied to phage preparations after propagation in bacterial hosts (Merabishvili et al., 2009), the success of which can then be confirmed on human cells using relevant exposure models. Previous investigations of this type have utilized immortal cell lines or animal models. Although the ability to screen the suitability of phage preparations using human CF airways cells would lead to more relevant findings, investigations involving primary airway epithelial cells have traditionally been limited due to their restricted expansion potential (Lane et al., 2005). However, our laboratory has recently described the development of an *in vitro* culture expansion methodology of primary airway epithelial cells in a realistic model of the airway (Martinovich et al., 2017). Using primary airway cells derived utilizing this technique, we performed screening analyses of a number of phages. Due to its broad range of activity against CF clinical isolates, phage E79 was purified using ion-exchange chromatography and endotoxin depleted to clinically acceptable levels. We showed that purified E79 does not induce apoptosis or inflammatory cytokine production in primary airway cells derived from children with CF or healthy non-CF individuals exposed to the phage preparations for 48 h. Moreover, the phage was virulent, stable, and genome sequencing did not reveal any known bacterial virulence genes. Collectively, these findings suggest that E79 may be a good candidate for phage therapy.

## Materials and Methods

### Bacteriophage and Bacterial Sources and Culture

E79 and F116 bacteriophages (Slayter et al., 1964) were obtained from the University of Western Australia Department of Microbiology Culture Collection (MCC), and utilized as examples of virulent and temperate phages to compare to novel phage isolates. Novel *P. aeruginosa* phage P5 was isolated from pediatric CF patient sputum. A panel of 21 isolates of *P. aeruginosa* (designated PMH-) were derived from sputum of patients with CF-related infections, supplied by PathWest Laboratory Medicine, Perth, Western Australia. An independent collection of 14 isolates of *P. aeruginosa* isolated from sputa of children with CF were derived from the AREST CF culture collection (designated ARESTCF-). Reference strain PAO1 (Dunn and Holloway, 1971) was obtained from MCC. *P. aeruginosa* strains were routinely propagated in heart infusion agar (HIA) or broth (HIB) aerobically at 37°C.

### Antimicrobial Susceptibility of Clinical *P. aeruginosa* Isolates

Sensitivity of 14 clinical isolates (AREST CF collection) of *P. aeruginosa* to gentamicin, ceftazidime, meropenem, amikacin, piperacillin, cefepime, tobramycin, ciprofloxacin, norfloxacin, timentin, piperacillin/tazobactam, and aztreonam was determined by the Royal Children’s Hospital Melbourne pathology laboratory using disk diffusion assays. Susceptibility was determined according to Clinical and Laboratory Standards Institute (CLSI) breakpoints (CLSI, 2016).

### Isolation of Clinically Derived Phages

Sputa from 20 CF patients were screened for the presence of phages that were able to infect any member of the panel of 21 clinical *P. aeruginosa* isolates (PMH collection). Sputa were mixed, and large materials sedimented by centrifugation at 1,750 ×*g* for 20 min at room temperature (RT). Following filter-sterilization of the supernatant using a 0.22 μm pore sterile membrane, an aliquot (10 μL) was pipetted onto a lawn of each bacterial strain as described for phage propagation. Plaques appearing on bacterial lawns, either derived from sputa or from lysogenic phage spontaneously induced from the bacterial genome, were transferred to 150 μL of phage buffer (Tris–HCl 10 mM, MgCl_2_ 10 mM [pH 8]) (Matsumoto et al., 1986). The suspension was purified as described for phage propagation and tested for phage titre. Phages were plaque-purified twice, prepared at high titre and stored at 4°C.

### Phage Imaging and Classification

Electron microscopy was performed on phage suspensions that were purified using a modified methodology (Bruttin and Brussow, 2005). Briefly, following centrifugation at 4,000 ×*g* for 15 min at RT the resulting supernatant was separated by centrifugation at 35,000 ×*g* for 25 min at RT. The phage-containing pellet was resuspended and applied to carbon-coated formvar grids for 5 min, which were charged prior to phage co-incubation using 1% (w/v) Alcian blue. Grids were negatively stained for 10 min using a 2% sodium silicotungstate solution, dried at RT, then analyzed using Transmission Electron Microscopy (TEM) using a Philips 401 TE microscope. Phage head, tail, and sheath sizes were measured, and data presented as a mean and standard deviation of three particle measurements.

For classification, phage genetic material was treated with the class II restriction endonucleases, either *Bam*HI or *Hind*III, for 2 h at 37°C. A 2 μL sample was then combined with 3 μL of tracking dye containing bromophenol blue (2.5 mg/mL) and sucrose (400 mg/mL) and electrophoresed on a 0.8% (w/v) agarose gel containing ethidium bromide (10 μg/mL) in Tris-Acetate Buffer with Ethylenediaminetetraacetic acid (0.5 M) for 60 min at 90 V. The gel was then photographed using UV illumination to detect if multiple bands were present, indicating digested DNA. The genome size was estimated by comparison to a *Hind*III ladder, while the genome sequencing results provided a more precise genome size.

### Phage Propagation and Host Range

Phages were propagated to high titre according to a modified version of the double agar layer method (Adams, 1959). Molten overlay agar containing 1% Bacto agar and HIB, supplemented with calcium and magnesium (0.01 M) was mixed with an aliquot of overnight broth culture of host bacteria and phage at approximately 10^5^ PFU/mL. The mixture was overlaid onto a HIA plate, allowed to set at RT and then incubated overnight at 37°C in 5% CO_2_. The following day, the overlay agar with semi-confluent phage plaques was scraped off the HIA plate and mixed with 3.5 mL of isotonic saline. All phage preparations were preliminarily purified by mixing followed by centrifugation at 1,750 ×*g* at RT. The supernatant was filtered through a 0.22 μm pore-size membrane. Phage titre was then determined using the drop-on plate method. Briefly, 10 μL of 10-fold dilutions of phage preparations were dropped in triplicate onto a HIA plate overlaid with overlay agar containing an overnight culture of PAO1, and the plaques in the bacterial lawn counted after incubation overnight at 37°C in 5% CO_2_.

Host range experiments were carried out on 21 clinical *P. aeruginosa* strains. Sensitivity to a phage was determined by a zone of clearing or plaques on a bacterial lawn where 10 μL of high titre phage preparation (10^8^–10^12^ PFU/mL) was spotted onto the lawn in triplicate, followed by incubation at 37°C overnight.

### Growth Inhibition by E79

The inhibitory capacity of E79 for clinical *P. aeruginosa* isolates (AREST CF collection) was determined using the following method. Briefly, bacteria were grown to approximately 0.5 MacPharland standard (∼1 × 10^8^ CFU/mL,) determined by spectroscopy and 100 μL of bacterial suspension was then added to wells of a 96-well flat-bottom polypropylene plate. E79 was diluted to 10^9^ PFU/mL in sterile isotonic saline, then serially diluted in 10-fold dilutions down to 10^4^ PFU/mL. Dilutions of phage or a control of saline only were added in equal volumes at a multiplicity of infection (MOI) of 10:1 and 1:1, and plates incubated at 37°C in 5% CO_2_ overnight. Optical density of wells was then measured at 595 nm, and the percentage of growth in treated wells quantified by dividing this value by the absorbance in the control well. A bacterial strain was considered sensitive to phage at that MOI if the optical density in the test well was ≥10% lower than that of the untreated well.

### Phage DNA Extraction and Genome Sequencing

High titre E79 was pre-treated with DNase I to reduce contaminating bacterial DNA. A 200 μL aliquot containing ∼10^11^ PFU of phage was incubated for 30 min at 37°C with 1 U of DNase I from the Invitrogen PureLink RNA Mini Kit. DNA was subsequently extracted using a QIAamp DNA mini spin column kit (QIAGEN) per manufacturer’s instructions and sequenced (LotteryWest Sequencing Facility, University of Western Australia, Perth, WA, Australia). The concentration of DNA was determined using a Qubit fluorometer according to manufacturer’s instructions. The sample (10 ng) was sheared using a S2 ultrasonicator (Covaris) and sequencing libraries prepared using a NEBnext Ultra library kit (New England Biosciences). Sequencing was performed on a 318 chip using an Ion Torrent PGM semiconductor sequencer for 820 flows. Data were collected using Torrent Suite 5.0. Reads were imported into CLC Genomics Workbench V 10.1.2, and *de novo* assembled, before the phage genome was annotated using RASTtk (Brettin et al., 2015). The annotated phage genome was then imported into Geneious V10.2.4 for visualization and manual curation.

### Purification of High Titre Phage Preparation for Cellular Assays

Filter-sterilized high titre phage preparations were purified using a CIMmultus quaternary amine (QA) advanced composite monolithic column (BIA separations) in combination with a Bio-Rad Econopump. Phage was diluted 1:10 in 20 mM Tris–HCl (pH 7.5) loading buffer, and 10 mL was passed through the column. The column was washed with 20 volumes of loading buffer, and then 20 mM Tris–HCl supplemented with successive increases of saline (250 mM–1.2 M NaCl) in 2 mL volumes, which were collected as separate fractions. The titre of phage in each fraction was tested as previously described and the fraction with the highest titre of phage selected for further purification through endotoxin-depletion.

Endotoxin was removed from the purified phage preparation using the EndoTrap HD 5/1 kit (Hyglos) per the manufacturer’s protocol. Here, phage preparation was diluted 1:5 in 20 mM Tris–HCl containing 450 mM NaCl and 0.125 mM CaCl_2_, passing the phage solution three times through the column. Concentrations of endotoxin in the phage preparations before and after purification were determined using the Endpoint Chromogenic Limulus Amebocyte Lysate (LAL) assay (Lonza), using endotoxin-free reagents and equipment. A high titre phage preparation (10^11^ PFU/mL), generated as previously described, was tested diluted in endotoxin-free water diluted 10^-8^ (10^3^ PFU/mL) to reach the detectable range of the kit. Purified phage preparations were tested in duplicate at 1:5 dilution in endotoxin-free water and the concentration interpolated from a standard curve using a 3PL curve fit in GraphPad Prism software.

### Study Participants and Epithelial Cell Collection

This study was carried out in accordance with the recommendations of the National Health and Medical Research Council of Australia’s National Statement on Ethical Conduct in Human Research with written informed consent was obtained from the parent or guardian of all participants. All participants gave written informed consent in accordance with the Declaration of Helsinki. The study was approved by St. John of God Health Care Human Research Ethics Committee Ref#901 sub-study 901.1050 and by the Princess Margaret Hospital for Children Ethics Committee Ref#1762EPP. Sixteen individual cell cultures derived from children with CF (*n* = 8) and from healthy non-CF children (*n* = 8), aged from 0–6 years were collected. Median ages of participants with or without CF were not significantly different (3.0 vs. 3.2 years old, respectively; *p* = 0.88), nor proportions of females in each of these groups (37.5% vs. 50%, respectively; *p* = 0.60). Parental consent was obtained prior to bronchial brushing and cells collected as described previously (Kicic et al., 2006; Sutanto et al., 2011). Epithelial cells were purified by removal of CD68+ cells and the epithelial phenotype confirmed (Lane et al., 2005; Kicic et al., 2006; McNamara et al., 2008). Demographics of the study cohort are shown in Table 1.

**Table 1:** Clinical details of CF-related characteristics in cell donors with CF at time of cell collection.

Sample	CFTR genotype	CFTR mutation class	Neutrophil elastase (nM)	Bronchiectasis (0–12 score)	Respiratory pathogens
CF1	p.Gly85Glu/Unknown	–/–	100	0	Mixed oral flora
CF2	p.Phe508del/p.Phe508del	II/II	1260	n/a	None
CF3	p.Phe508del/p.Phe508del	II/II	270	n/a	*Moraxella catarrhalis*, *Haemophilus influenzae,* MRSA, Mixed oral flora
CF4	p.Phe508del/p.Asn1303Lys	II/II	270	n/a	*Aspergillus fumigatus*, *Stenotrophomonas maltophilia*
CF5	p.Phe508del/p.Thr966ArgfsX2	#N/A	170	n/a	None
CF6	p.Phe508del/Ala455Glu	#N/A	70	n/a	*H. influenzae*
CF7	p.Phe508del/p.Phe508del	II/II	140	n/a	*Streptococcus pneumoniae*
CF8	p.Phe508del/p.Phe508del	II/II	60	n/a	None

*CF, cystic fibrosis; CFTR, cystic fibrosis transmembrane conductance regulator; MRSA, methicillin-resistant *Staphylococcus aureus*.*

### Cell Cultures

Children with CF had cell samples collected during bronchoscopy as part of their annual clinical surveillance program (Sutanto et al., 2011). In addition, cells from non-CF controls were collected from children during elective non-respiratory related surgery where intubation was required.

**FIGURE 1:**
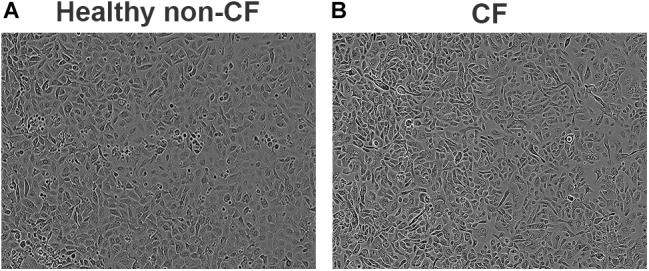
Phase contrast images of confluent primary airways cell cultures obtained using conditionally reprogrammed method. Epithelial cobblestone morphology is typically maintained and observed in both healthy non-CF **(A)** and CF **(B)** primary cell cultures. Representative image of eight subjects per phenotype. Magnification 100×.

Primary airway epithelial cells derived from bronchial brushings were conditionally reprogrammed, a method which we previously reported resulted in cells that retain their phenotype and functionality in culture (Martinovich et al., 2017). Briefly, airway epithelial cells were co-cultured with irradiated fibroblasts and grown on tissue culture-grade flasks that were pre-coated with fibronectin and type I collagen (Kicic et al., 2006). Cells were grown to 90% confluence in pre-coated flasks in culture media for approximately 5 days, and then seeded into 96-well plates at ∼20,000–40,000 cells/well in growth media containing bronchial epithelial basal medium (BEBM^®^; Lonza, Basel, Switzerland) supplemented with SingleQuot additives. All cell cultures were at passage 3 at time of experiments and were maintained at 37°C in an atmosphere of 5% CO_2_ under aseptic conditions. Experiments were conducted on multi-layered primary airway epithelial submerged cultures which exhibit typical cobblestone morphology (Figure 1).

### Stimulation of Cell Cultures

Prior to exposure to phage, media in 96-well plates was changed to starvation media (growth medium minus bovine pituitary extract, epidermal growth factor, and antibiotics) for 24 h. Cells were then exposed to saline control or phage treatments in infection media (media minus bovine pituitary extract and antibiotics). The following treatments were applied to cells as 10% of the medium in 90% v/v infection media: (1) isotonic saline, (2) E79 phage (ratio 1:1 per epithelial cell), and (3) E79 (ratio of 10:1 per epithelial cell). Cells were then incubated for 48 h at 37°C in 5% CO_2_. After exposure, cell supernatant was collected from replicate wells, pooled and frozen at -80°C until batch cytokine analyses were performed. Following removal of supernatant from 96-well plates, cell viability was assessed.

### Cytokine Detection in Cell Culture Supernatants

The concentration of IL-8 in culture supernatants was determined using enzyme-linked immunosorbent assays (ELISA; R&D Systems) previously used in our laboratory (Kicic et al., 2016). Values were interpolated from a standard curve with a range of 3.125–200 pg/mL of IL-8. IL-6 was measured in culture supernatants using a time-resolved fluorescence immunoassay as previously described (Taylor et al., 2007; Sutanto et al., 2011) with a minimum detectable value of 3 pg/mL. The concentration of IL-1β in cell culture supernatants was tested using commercially available ELISA kits, according to the manufacturer instructions (eBioscience), with a limit of detection of 2 pg/mL. Fluorescence and absorbance measurements for immunoassays were detected using the ClarioStar plate reader (software version 5.20 R5 from BMG Labtech). Concentrations of cytokines were interpolated from measurements using a standard curve used in the same assay. Data were transformed using the calculation x = log(x) and a 4-parameter logistic curve fit in GraphPad Prism software. Any values falling below the limit of detection were assigned a value of half the lowest detectable concentration multiplied by the dilution factor. Following interpolation of concentrations, they were back-transformed using *x* = 10*^x^*.

### Cell Viability and Apoptosis Measurements

Airway epithelial cell viability was determined using the CellTiter 96 Aqueous Non-Radioactive Cell Proliferation Assay Kit (Promega). Following incubation as instructed, absorbance of each well was measured at 492 nm using a plate reader. A control well without cells was used to determine background absorbance. The percentage viability in each treatment well was then compared to the calculated mean control value. Mean values from at least three technical replicate wells were used in final calculations.

Apoptosis was measured in cells exposed to phage for 48 h using a ssDNA apoptosis ELISA Kit (Merck Millipore) as previously described (Sutanto et al., 2011; Kicic et al., 2016). Briefly, following exposure, culture supernatant was removed, and cells were fixed using 200 μL per well of a solution containing 80% methanol and 20% PBS. Plates were then air dried for 30 min at RT and stored at 4°C until batch analyses of apoptosis were performed. The percentage of apoptotic cells compared to untreated controls was then determined where data were normalized to the control apoptosis value for that plate.

### Statistical Analyses

Continuous variables were tested for normality using a Shapiro–Wilk test. Since most variables did not follow a normal distribution, non-parametric tests were used. Categorical variables in the CF and healthy non-CF cohorts were compared using a Chi-squared test, and continuous clinical variables were compared using a Mann–Whitney test. Cytokine measurements were presented as both absolute concentrations measured in supernatants adjusted for viable cell numbers, and for the purposes of comparing between the CF and healthy non-CF groups, cytokine levels in phage treated wells were normalized to the saline well as a ratio of 1. Paired data including results from cell culture experiments and bacterial minimum inhibitory concentration assays were compared using a Friedman test or Wilcoxon signed-rank test, comparing phage treatments to the control (saline), with Dunn’s post-test applied for multiple comparisons where appropriate. Unpaired data comparing cytokine fold-change in phage-treated wells between CF and HNA were compared using Mann–Whitney tests. All statistical analyses were performed in SPSS v24 (IBM). In all tests, a *p*-value <0.05 was considered statistically significant.

## Results

### Isolation of Clinically Derived Phage

One *P. aeruginosa* phage (P5) was successfully isolated from patient sputum by direct plating and propagated on patient strain PMH5. Six other phages were observed as plaques on lawns of bacterial isolates derived from CF patients, but could not be successfully propagated to high titre or were unstable at 4°C in storage, and thus were not investigated further. In total, 6/20 (30%) CF-derived *P. aeruginosa* were demonstrated to carry spontaneously inducible lysogenic phages.

### Phage Host Ranges

Plaque formation by E79 was observed on lawns of 20/22 (91%) of *P. aeruginosa* strains tested (Table 2), making it a strong candidate for further testing as a potential therapeutic phage. F116 and P5 had more limited host ranges (36 and 64% strains susceptible, respectively), and were known (F116) or suspected (P5) temperate phages, and were therefore eliminated from further screening, since these were not considered to be ideal for phage therapy.

**Table 2:** Host range of phages within the panel of clinical *P. aeruginosa* isolates and PAO1 strain.

*P. aeruginosa* strain	Colony phenotype	Phage susceptibility		
		E79	F116	P5
PAO1	Smooth	++	++	++
PMH1	Mucoid	++	–	++
PMH2	Smooth	++	–	–
PMH3	Mucoid	++	–	–
PMH4	Mucoid	++	–	+
PMH5	Smooth	++	+	++
PMH6	Rough	++	–	–
PMH7	Mucoid	++	–	+
PMH9	Smooth	–	–	++
PMH10	Mucoid	–	–	++
PMH11	Rough	++	+	++
PMH12	Mucoid	++	–	–
PMH13	Smooth	++	+	++
PMH14	Smooth	++	–	–
PMH15	Mucoid	++	–	–
PMH16	Rough	++	–	–
PMH17	Smooth	++	–	+
PMH18	Rough	++	–	–
PMH19	Mucoid	++	+	+
PMH20	Mucoid	++	++	++
PMH22	Mucoid	++	+	+
PMH23	Rough	++	+	+

*++Indicates complete clearing, +indicates partial clearing, –indicates no visible clearing of the bacterial lawn.*

### E79 Genetic and Morphological Characterization

Both E79 and P5 were confirmed to be double-stranded DNA phages, demonstrated by digestion by *Hind*III and *Bam*HI, respectively (not shown). TEM of E79 identified the phage to have a head diameter of 63.6 ± 3.8 nm, head length of 60.5 ± 5.7 nm, tail width of 8.4 ± 0.8 nm, tail length of 128.1 ± 3.7 nm, and a sheath width of 16.6 ± 2.4 nm. Genome sequencing of E79 generated 2.2 M reads with an average length of 252 bp. The sequencing analysis returned a complete genome of 66,061 bp, with BLASTN analysis revealing 98% query cover and 97% identity to a range of widespread PB1-like *Pseudomonas* phages in the *Myoviridae* family (Ceyssens et al., 2009). The E79 genome contained 96 open reading frames, 75 of which were for hypothetical phage proteins. No virulence factors or antibiotic resistance genes were detected, nor any genes from the host PAO1 bacterial genome. The full sequence of E79 is deposited in GenBank under accession #MH536736.

### Phage Purification

**FIGURE 2:**
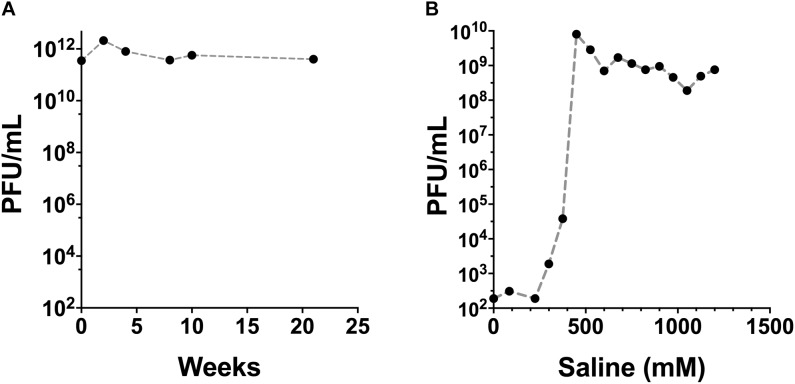
Stability and recovery of E79 phage after purification steps. **(A)** Titre of E79 phage was retained over long term storage at 4°C, determined by the drop-on-plate method using PAO1 as the propagating host strain. **(B)** Recovery of E79 in fractions from ion-exchange chromatography column. Data show average values obtained from three replicate measurements of a single solution.

Phage fractions were collected from the ion-exchange chromatography column and examined for remaining viable phage. Approximately 80% of the added phage was recovered in the saline fractions collected. Maximal phage recovery occurred from the elution at approximately 450 mM NaCl (Figure 2B). The concentration of endotoxin in the filter-sterilized phage (unpurified) and the purified, endotoxin-depleted phage preparations was approximately 10^8^ and 1.9 EU/mL, respectively. E79 phage preparations were stored at 4°C and retained the high infective titre for >20 weeks of testing (Figure 2A).

### Bacterial Growth Inhibition

Growth of clinical *P. aeruginosa* isolates was significantly decreased (median 33% decrease) in the MOI 1:1 (low phage ratio) and MOI of 10:1 (high phage ratio) (median 23% decrease). Of 14 clinical isolates tested, a variety of growth inhibition and clinical antibiotic resistance phenotypes were observed (Table 3) with 11 (79%) showing some growth inhibition by E79. Of these, eight (57%) exhibited single or multiple antibiotic-resistance.

**Table 3:** Comparative sensitivity of clinical *P. aeruginosa* isolates to E79 phage at high MOI (10:1 ratio to bacterial cells) or low MOI (1:1 ratio to bacterial cells) and to antibiotics.

Sample ID	Gentamicin	Ceftazidime	Meropenem	Amikacin	Piperacillin	Cefepime	Tobramycin	Ciprofloxacin	Norfloxacin	Timentin	Piperacillin/ Tazobactam	Aztreonam	E79 at low MOI	E79 at high MOI
ARESTCF-1	S	S	S	S	S	S	S	S	S	**I**	S	S	S	S
ARESTCF-2	S	S	S	S	S	S	S	S	S	**I**	S	**I**	**R**	S
ARESTCF-3	S	S	S	S	S	S	S	S	S	S	S	**I**	S	S
ARESTCF-4	S	S	S	S	S	S	S	S	S	S	S	S	S	S
ARESTCF-5	S	S	S	S	S	S	S	S	S	**I**	S	S	S	S
ARESTCF-6	S	S	S	S	S	S	S	S	S	**I**	S	S	S	S
ARESTCF-7	S	S	S	S	S	S	S	S	S	**R**	S	**R**	**R**	**R**
ARESTCF-8	S	S	S	S	S	S	S	S	S	S	S	S	S	S
ARESTCF-9	S	S	S	S	S	S	S	S	S	S	S	S	**R**	**R**
ARESTCF-10	S	S	S	S	S	S	S	S	S	S	S	S	S	S
ARESTCF-11	S	S	S	S	S	S	S	S	S	S	S	**R**	S	S
ARESTCF-12	S	S	S	S	S	S	S	S	S	S	S	S	**R**	**R**
ARESTCF-13	**I**	S	S	S	S	S	S	S	S	S	S	S	**R**	S
ARESTCF-14	S	S	S	S	S	S	S	S	S	S	S	S	S	S

*MOI, multiplicity of infection; S, sensitive; I, intermediate; R, resistant. Results with intermediate resistance or resistance are indicated with bolded font and gray shading.*

### Response of Airway Epithelial Cells to Stimulation

**FIGURE 3:**
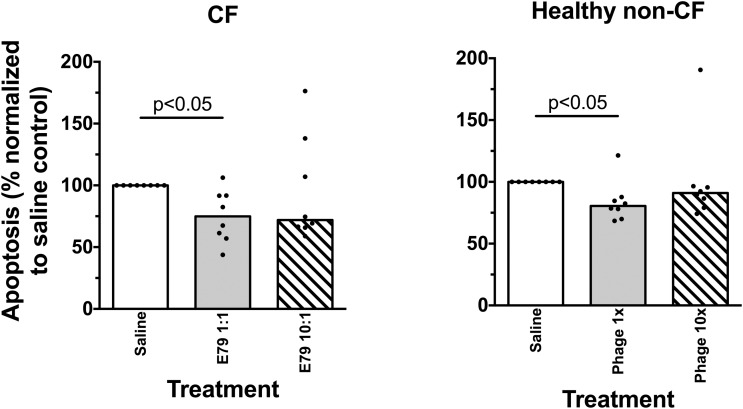
Survival of primary airways cells exposed to phage. Apoptosis in cell cultures exposed to phage at high or low concentration relative to saline controls. Average individual values are shown from three replicate wells, and bars indicate the group median value. Statistically significant differences in Friedman post-tests (*p* < 0.05) are indicated by *p*-values.

Viability of airway epithelial cells from children with CF or healthy non-CF controls was not affected when treated with phage at either ratio (data not shown). Similarly, E79 phage did not induce apoptosis in either phenotype at either the low or high phage ratio (Figure 3); rather exposure to phage was associated with lower apoptosis levels than observed in control wells.

**FIGURE 4:**
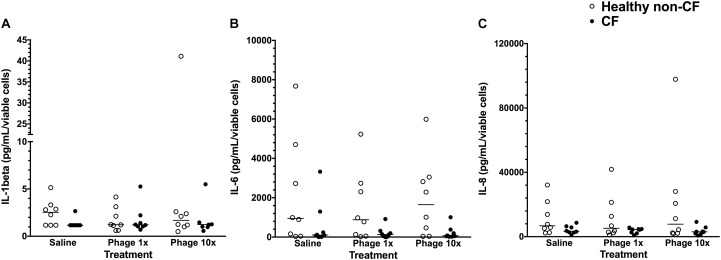
Cytokine production in primary airways cells exposed to phage. Levels of **(A)** IL-1β, **(B)** IL-6, and **(C)** IL-8 cytokines in culture supernatants of cells exposed to treatments for 48 h compared with untreated controls cell. Cytokine concentrations were adjusted for the ratio of viable cells in the phage treatment wells compared with the control well. Data show values for healthy non-CF children (open circles; *n* = 8) or children with CF (closed circles; *n* = 8) representing the mean calculated from three replicate treatment wells per individual. Horizontal lines represent the group (healthy non-CF or CF) median.

Comparing 8 non-CF and 8 CF cultures treated with phage suspensions at low or high ratio, there was no induced inflammatory cytokine production (Figure 4). Specifically, no significant increase in IL-1β, IL-6, and Il-8 was observed when CF or healthy non-CF cells were treated with low or high phage titres (*p* > 0.05; Figure 4).

## Discussion

New antibacterial therapies are urgently required to counteract the rise of multidrug-resistant microbes. In this work, we successfully propagated and screened three bacteriophages and identified that the *P. aeruginosa-*infecting phage E79 had the broadest host range of these. Moreover, the protocol utilized to purify phage was successful in removing bacterial contaminants to the extent that no significant apoptosis, or inflammatory cytokine production was detected in either airway epithelial cells from children with CF or healthy non-CF controls after 48 h of co-culture with purified E79 phage. Collectively, this work demonstrates a proof of concept that a screening approach utilizing bacteriophage and primary epithelial cells derived from the CF population could be applied for the development of new therapies for people with CF-associated antibiotic-resistant bacterial lung infections.

Given the association of *P. aeruginosa* airway infections and pulmonary decline in people with CF, early intervention when children are first infected could be critical in preventing lung damage and increase life expectancy (Ranganathan et al., 2017). Inhaled phage therapy is one option that could be utilized in combination with current therapies to improve outcomes. The optimal dosing formulations and regimes would need to be determined in clinical trials following identification of ideal phage candidates, using pre-clinical models such as the one described here. A limited number of case studies are described in the literature on treatment of airways infections in children with CF with phage therapy, as well as a number of reports on use in uncontrolled studies in CF and non-CF adults (Sulakvelidze et al., 2001; Kutateladze and Adamia, 2008; Kvachadze et al., 2011; Abedon, 2015; Hoyle et al., 2018). Encouragingly, these early reports suggest that phage therapy may be feasible in the pediatric CF population.

Our pre-clinical screening method demonstrated that almost half of *P. aeruginosa* isolates carried one or more spontaneously released phages. It is likely that these phages were temperate, and therefore may not be ideal for therapeutic use (Hraiech et al., 2015), although we did not confirm this with genome sequencing. Isolation and propagation to high titre proved difficult for most of these phages. The prevalence of non-propagatable isolates in this study is not unique (Serwer et al., 2004), and isolation techniques could be optimized in future, such as inclusion of antibiotics in culture media (Santos et al., 2009). Despite the potential for lysogenic phages to confer resistance to related phages (Labrie et al., 2010; James et al., 2012; van Houte et al., 2016), each bacterial isolate utilized for screening of host ranges here was susceptible to at least one phage tested for activity, indicating that the children from whom the bacteria were isolated could potentially be treated with phage therapy.

We found that the virulent phage, E79, had many ideal characteristics for further development as a therapeutic phage, including stability in high titre preparation, broad infective capacity of clinical *P. aeruginosa* isolates, and lack of known bacterial pathogenicity genes. Indeed, PB1-like phages such as E79 are considered by some to be among the most promising for application in phage therapy (Krylov et al., 2015) due in part to the low frequency of mutation to phage-resistance (Ceyssens et al., 2009). On the other hand, an earlier study suggested that E79 had restricted access to the deeper layers of a biofilm of PAO1 generated on glass (Doolittle et al., 1996), although the authors stated that fluorescently labeling the phage significantly affected infective capacity and that they could not determine whether the decrease in signal deeper in the biofilm was an experimental artifact. In another study, biofilm challenge with E79 resulted in a transient increase at 24 h in biofilm formation, although following further incubation, biofilm decreased (Hosseinidoust et al., 2013). Despite the limitations of extrapolating artificial biofilm methods to human CF lungs, which have very different environmental conditions that might affect bacterial growth, these are important issues to address for phage therapy. Certainly, further characterization would be required for clinical development in a more disease-appropriate model system. Here, to determine bacterial infective capacity, we used a qualitative drop-on-plate assay and an indirect optical density assay of bacterial viability. Whilst these assays may give some indication of a phage’s broad infective capacity of various bacterial strains, more accurate prediction of clinical outcomes in patients would ideally involve quantitative measurement of phage activity in a biofilm model, ideally including live/dead cell imaging.

Phage-host dynamics are complex with pharmacokinetic interactions that may not be correctly predicted without including as many components as possible of the phage-bacterial-host cell interaction in a model. *In vivo*, it is predicted that the human immune system would work in a complementary manner to phage to control bacterial growth. Roach et al. (2017) reported that neutrophils are important for immunophage synergy and immunodeficient mice did not respond to phage treatment of *P. aeruginosa* pneumonia in the same manner as immunocompetent mice. Therefore, simple antimicrobial assays may not accurately predict clinical consequences of novel treatments. Given the differences between healthy and CF neutrophil phenotypes (Gray et al., 2018), it is critical to examine immunophage synergy for people with CF in a relevant model for these individuals, and as such it may be possible to include neutrophils or other immune cells in an airways co-culture model. Although we did not include any non-epithelial cells in this model, one major focus was adequate purification of phage preparation. Given that human airways epithelial cells express a range of Toll-like receptors (Ritter et al., 2005), and our work assessed upregulation of downstream cytokines from NF-κB activation, our model was sufficient for this aim. The response of primary cells derived from different children was personalized; though overall, phage preparations did not induce cytokine production, in some individuals, production of inflammatory cytokines in response to phage exposure was elevated when compared to untreated cells. Despite this, as a group (CF or non-CF children) the differences between phage-exposed and control cell cultures were not statistically significant. Therefore, our data suggest that personalized screening approaches may be best to determine the appropriate treatment for people with CF, where grouped results may not be representative of outcomes for all individuals within that group. However, the method used here in this pilot study could be significantly improved in future by one or all of the following: (a) use of air-liquid interface (ALI) in primary respiratory cell cultures, (b) examination of biofilm, (c) addition of professional immune cells in co-culture with respiratory cells, and (d) delivery of aerosolised phage preparations.

The phage purification methods used here were comparable to those used successfully by others, and resulted in depletion of endotoxin and other bacterial signatures that might have activated epithelial cell inflammatory responses. However, it is likely that without extra preparatory steps, host DNA contamination may be an issue for phage preparations (Kleiner et al., 2015). In addition, it is possible that the decreased levels of apoptosis in phage suspension-treated cells compared with saline-exposed cells is a host cell pro-survival response to damage caused by other PAO1 contaminants remaining in the phage preparation (Wu et al., 2011). Given that we did not find any PAO1-associated genes in the E79 genome, our methods to remove free host DNA were successful, and therefore, it may be appropriate to utilize a similar DNase treatment step to avoid potential transfer of host bacterial genes to bacteria in the human airways, as previously suggested (Pirnay et al., 2015). Previous work with other phages has shown different methods of pharmacological preparation affect viability and particle sizing, which are relevant for inhalation of phages (Leung et al., 2016). In the CF lung environment, it will be important to determine the pharmacological formulations that best allow phages to survive the preparation steps and to enter the lungs with optimal aerodynamic properties in an inhaled formulation (Chang et al., 2017). The activity of optimized phage formulations in the lung environment can be investigated using models of the CF airway, although more purification steps should be taken with phage preparations in future.

In this study, of eight clinical isolates of *P. aeruginosa* that displayed some level of resistance to single or multiple antibiotics, seven exhibited E79 phage-susceptibility at the highest MOI tested, while two out of six antibiotic-susceptible isolates were E79 phage-resistant. This preliminary result suggests that the combined use of traditional antibiotics and phages might be a good approach in at least some cases of antibiotic-resistant infections. Rossitto et al. (2018) have noted the limited number of investigations of combined antibiotic-phage treatment, but consider this a promising approach for the future of anti-*P. aeruginosa* therapy in CF. The rate of progress from bench to bedside that might be predicted by the ease with which novel phages are isolated is in fact limited by the uncertainty surrounding their safety. This situation arises, in part, from inadequate characterization of these phages in clinically relevant CF models. Therefore, in future, the process of selecting phages for therapeutic development could be performed as a high-throughput methodology for people with CF, identifying phages that are suitable for their particular infecting bacterial strain and immunological idiosyncrasies, and determining appropriate dosage using appropriate personalized *in vitro* models.

## Author Contributions

BC, ST, and AK developed the research concept and designed the experiments. ST and AK carried out the experimental work. ST and MO conducted the data analysis. ST wrote the first draft of the manuscript. All authors contributed to the drafting and editing of the manuscript.

## Conflict of Interest Statement

AK, SS, and ST have registered a provisional patent related to the use of primary airway cells as a screening tool (P104811.AU). The remaining authors declare that the research was conducted in the absence of any commercial or financial relationships that could be construed as a potential conflict of interest.

### Members of the WAERP

Anthony Kicic, Stephen M. Stick, Darryl A. Knight, Elizabeth Kicic-Starcevich, Luke W. Garratt, Marc Padros-Goosen, Ee-Lyn Tan, Erika N. Sutanto, Kevin Looi, Jessica Hillas, Thomas Iosifidis, Nicole C. Shaw, Samuel T. Montgomery, Kak-Ming Ling, Kelly M. Martinovich, Francis J. Lannigan, Ricardo Bergesio, Bernard Lee, Shyan Vijaya-Sekeran, Paul Swan, Mairead Heaney, Ian Forsyth, Tobias Schoep, Alexander Larcombe, Monica Hunter, Kate McGee, Nyssa Millington.

### Members of the Australian Respiratory Early Surveillance Team for Cystic Fibrosis (AREST CF)

The full membership of the Australian Respiratory Early Surveillance Team for Cystic Fibrosis (AREST CF) is available at www.arestcf.org.

### Members of the Australian Respiratory Epithelium Consortium (AusREC)

Anthony Kicic, Stephen M. Stick, Elizabeth Kicic-Starcevich, Luke W. Garratt, Erika N. Sutanto, Kevin Looi, Jessica Hillas, Thomas Iosifidis, Nicole C. Shaw, Samuel T. Montgomery, Kak-Ming Ling, Kelly M. Martinovich, Matthew W-P Poh, Daniel R. Laucirica, Craig Schofield, Samantha McLean, Katherine Landwehr, Emma de Jong, Nigel Farrow, Eugene Roscioli, David Parsons, Darryl A. Knight, Christopher Grainge, Andrew T. Reid, Su-Kim Loo, and Punnam C. Veerati.
